# Evaluation of a Machine Learning Model Based on Laboratory Parameters for the Prediction of Influenza A and B in Chongqing, China: Multicenter Model Development and Validation Study

**DOI:** 10.2196/67847

**Published:** 2025-05-15

**Authors:** Weiwei Hu, Yulong Liu, Jian Dong, Xuelian Peng, Chunyan Yang, Honglin Wang, Yong Chen, Shan Shi, Jin Li

**Affiliations:** 1 Department of Laboratory Medicine The Affiliated Dazu’s Hospital of Chongqing Medical University Chongqing China; 2 Department of Respiratory and Critical Care Medicine The First Affiliated Hospital of Chongqing Medical University Chongqing China

**Keywords:** routine blood parameters, machine learning, influenza A and B, CATB-based AI-Lab, multicenter validation

## Abstract

**Background:**

Influenza viruses are major pathogens responsible for acute respiratory infections in humans, which present with symptoms such as fever, cough, sore throat, muscle pain, and fatigue. While molecular diagnostics remain the gold standard, their limited accessibility in resource-poor settings underscores the need for rapid, cost-effective alternatives. Routine blood parameters offer promising predictive value but lack integration into intelligent diagnostic systems for influenza subtyping.

**Objective:**

This study aimed to develop a machine learning model using 24 routine blood parameters to predict influenza A and B infections and validate a deployable diagnostic tool for low-resource clinical settings.

**Methods:**

In this multicenter retrospective study, 6628 adult patients (internal cohort: n=2951; external validation: n=3677) diagnosed with influenza A virus infection (A+ group), influenza B virus infection (B+ group), or those presenting with influenza-like symptoms but testing negative for both viruses (A–/B– group) were enrolled from 3 hospitals between January 2023 and May 2024. The CatBoost (CATB) algorithm was optimized via 5-fold cross-validation and random grid search using 24 routine blood parameters. Model performance was evaluated using metrics such as the area under the curve (AUC), accuracy, specificity, sensitivity, positive predictive value, negative predictive value, and *F*_1_-score across internal testing and external validation cohorts, with Shapley additive explanations analysis identifying key biomarkers. The Artificial Intelligence Prediction of Influenza A and B (AI-Lab) tool was subsequently developed on the basis of the best-performing model.

**Results:**

In the internal testing cohort, 7 models (K-nearest neighbors, naïve Bayes, decision tree, random forest, extreme gradient boosting, gradient-boosting decision tree, and CatBoost) were evaluated. The AUC values for diagnosing influenza A ranged from 0.788 to 0.923, and those for influenza B from 0.672 to 0.863. The CATB-based AI-Lab model achieved superior performance in influenza A detection (AUC 0.923, 95% CI 0.897-0.947) and influenza B (AUC 0.863, 95% CI 0.814-0.911), significantly outperforming conventional models (K-nearest neighbors, RF, and XGBoost; all *P*<.001). During external validation, AI-Lab demonstrated high performance, achieving an accuracy of 0.913 for differentiating the A+ group from the A–/B– group and 0.939 for distinguishing the B+ group from the A–/B– group.

**Conclusions:**

The CATB-based AI-Lab tool demonstrated high diagnostic accuracy for influenza A and B subtyping using routine laboratory data, achieving performance comparable to rapid antigen testing. By enabling timely subtype differentiation without specialized equipment, this system addresses critical gaps in managing influenza outbreaks, particularly in resource-constrained regions.

## Introduction

Influenza is an acute respiratory infectious disease caused by influenza viruses, primarily transmitted through airborne routes such as droplets and aerosols [1-3]. This mode of transmission makes influenza highly contagious, often leading to large-scale outbreaks and pandemics globally. Global epidemiological data from the World Health Organization reveals that seasonal influenza affects approximately 5% to 10% of the adult population worldwide annually. This respiratory infection leads to 3-5 million cases of severe illness and causes between 290,000 and 650,000 respiratory-related fatalities each year. The influenza virus family comprises 4 distinct classifications (A, B, C, and D), with type A and B strains demonstrating the highest clinical relevance in human populations. These two predominant subtypes are principally responsible for triggering widespread seasonal epidemics and inducing substantial disease burden through their capacity for rapid antigenic variation and efficient human-to-human transmission [3-5]. In 2023, after the sudden relaxation of COVID-19 pandemic control measures, China experienced widespread outbreaks of influenza A and B in several regions [6]. Therefore, the early detection of warning signals for influenza A and B is crucial for effective prevention and treatment, carrying significant practical implications.

Early diagnostic methods for influenza include blood tests, viral nucleic acid testing, and viral antigen detection, with nasopharyngeal swab nucleic acid testing currently being the gold standard for diagnosis [7]. However, routine blood test is the quickest and most used method in clinical practice. Recent studies have shown that parameters obtained from routine blood tests have significant predictive and prognostic value for infectious diseases [8-10]. These tests can be quickly completed and are easily accessible in primary health care settings, offering a simple and cost-effective tool for differential diagnosis. Existing research has reported that machine learning models trained using routine blood parameters are increasingly being developed and used for predicting various infectious diseases and cancers, with artificial intelligence (AI) models demonstrating good specificity and accuracy [11-14]. Consequently, machine learning methods can be used to develop simple and economically intelligent models for screening and diagnosing influenza A and B. This study investigates the diagnostic potential of routine hematological indicators for early-stage detection of influenza A and influenza B infections through the development of machine learning–based predictive models.

## Methods

### Study Design and Participants

This multicenter retrospective study enrolled 6628 adult patients (aged 18 years or older; internal cohort: 2951 patients; external validation cohort: 3,677 patients) diagnosed with influenza A virus infection (A+ group), influenza B virus infection (B+ group), or influenza-like symptoms with negative reverse transcriptase–polymerase chain reaction (RT-PCR) results for both viruses (A–/B– group). Patients were recruited from 3 hospitals: the Affiliated Dazu’s Hospital of Chongqing Medical University (cohort 1), the Affiliated Bishan’s Hospital of Chongqing Medical University (cohort 2), and the First Affiliated Hospital of Chongqing Medical University (cohort 3), between January 2023 and May 2024. Patient demographics and preexisting health conditions are summarized in Figure S1 in Multimedia Appendix 1. Blood test and RT-PCR results of the included patients were obtained within 24 hours. For patients with multiple blood tests within 24 hours, only the test closest to the RT-PCR sampling time was selected. Patients who received antiviral treatment prior to blood sampling or had duplicate records (identified using unique national identification numbers) were excluded.

A total of 27 routine blood parameters were collected during the initial disease evaluation, corresponding to the first available blood results. Patients who received any antiviral treatment prior to blood sampling were excluded. To prevent sample leakage across multiple centers, we excluded duplicate patients by using unique national identification numbers after data collection. To ensure an accurate representation of the patient’s current condition, the interval between blood sample and RT-PCR sample collection was typically kept within 12 hours and did not exceed 24 hours. For patients undergoing multiple blood tests within 24 hours, only the test closest to the RT-PCR sampling time was selected. For model development, hematological parameters containing more than 25% missing observations in any dataset were excluded through preliminary screening. This quality control process yielded 24 clinically relevant variables for subsequent analysis: age (years) along with cellular blood components including white blood cell count (10^9^/L), red blood cell count (10^12^ /L), hemoglobin (g/L), hematocrit (%), mean corpuscular volume (fL), mean corpuscular hemoglobin (pg), mean corpuscular hemoglobin concentration (g/L), platelet count (10^9^/L), platelet distribution width (%), mean platelet volume (fL), plateletcrit (%), neutrophil ratio (%), lymphocyte ratio (LYMPH%, %), monocyte ratio (MONO%, %), eosinophil ratio (EOS%, %), basophil ratio (BASO%, %), neutrophil absolute count (10^9^/L), lymphocyte absolute count (LYMPH#, 10^9^/L), monocyte absolute count (10^9^/L), eosinophil absolute count (EOS#, 10^9^/L), basophil absolute count (10^9^/L), red cell distribution width (%), and platelet-large cell ratio (%). For variables with less than 25% missing values, the commonly used K-nearest neighbors (KNN) imputation method was applied to complete the data.

For model training, we divided the patient data from 2 independent hospitals, cohort 1 and cohort 2, into training and internal test cohorts. Additionally, to further validate the generalization performance of the model, we collected laboratory test data from cohort 3 for external validation. This validation approach allows for a more accurate assessment of the model’s performance in real-world clinical settings, enhancing its stability and applicability across different datasets and clinical scenarios. The comparison of continuous variables was conducted using ANOVA, and all *P* values were adjusted using the false discovery rate method [15].

### Model Construction and Validation Process

In this research, we applied machine learning techniques to develop an auxiliary diagnostic system for influenza A and B based on routine blood parameters. The flowchart comprehensively outlines the entire study process (Figure S2 in Multimedia Appendix 1). The process includes data collection and AI diagnostics, where routine laboratory blood parameters are gathered to create a dataset, and influenza A and B ribonucleic acid are detected using RT-PCR. In the data preprocessing stage, the collected data are prepared for model training. Various machine learning methods are then used to build the models, and the best-performing model is selected. The model construction diagram details the comprehensive process of developing machine learning models (Figure S3 in Multimedia Appendix 1). Upon completion of model training, the trained model is implemented into a user-friendly interface, enabling physicians to quickly diagnose influenza A and B infections using routine blood parameters.

### Model Benchmarking

This study aimed to address a multinomial 2-class classification issue by distinguishing among patients with influenza A, influenza B, both A and B infections, and those presenting influenza-like symptoms but testing negative for both viruses. To facilitate the selection of preprocessing methods and benchmark algorithms, the 2-class classification problem was divided into 2 binary one-versus-all tasks. The algorithms evaluated included KNN, naïve Bayes (NB), decision tree (DT), random forest (RF), extreme gradient boosting (XGBoost), gradient-boosting decision tree (GBDT), and CatBoost (CATB). This diverse selection allowed for a comparison of 7 different algorithmic designs.

For each model, optimal parameters were determined through a stratified 5-fold cross-validation on the training set, using a random grid search for hyperparameter tuning using the Scikit-learn Python package (Table S1 in Multimedia Appendix 1). Model performance was then assessed on the internal testing set, using the area under the curve (AUC) and its 95% CI, based on 2000 nonparametric bootstraps. The impact of various data preprocessing methods was analyzed for each algorithm across 23 variables. Missing-value imputation was conducted using the Scikit-learn Python package. All imputation and scaling parameters were derived from the training set and subsequently applied to the internal testing set. All analyses were executed using Python (version 3.11; Python Software Foundation).

### Performance of Different Models

Bootstrapping is widely recognized as a powerful resampling method in modern statistics, used to estimate the distribution of statistics (such as mean, variance, SD, etc) by repeatedly sampling the original data with replacement [16]. To impartially evaluate the classification performance of various models and safeguard the model’s generalization capability from the impact of data division methods, we use Bootstrapping to randomly sample the dataset 2000 times. This process aimed to calculate the mean and CIs of the 7 metrics, comprehensively assessing the model’s generalization ability.

### Implementation of Graphical Interface and Web Application

Artificial Intelligence Prediction of Influenza A and B (AI-Lab) incorporates a user-friendly graphical interface developed using the open-source Python package Streamlit. The entire application is accessible via a web-based interface, making it easy for health care professionals to use the machine learning models developed for clinical diagnosis. AI-Lab offers 3 methods for data entry, which are automatic entry, manual entry, and batch entry. The automatic entry function retrieves basic patient information and laboratory results directly from the hospital information system using the patient’s ID number. While this feature requires customization for each medical center’s hospital information system, it provides a highly automated, user-friendly, and efficient screening process. Manual entry allows users to input basic patient information and lab results manually, making it ideal for single diagnoses. Batch entry facilitates bulk screening by uploading patient information into a prespecified template file. Once data are uploaded, the system automatically checks their validity. If the data is valid, all subsequent processing and calculations are completed without further user intervention. The streamlined operation and clear workflow make AI-Lab particularly effective for rapid screening of conditions such as influenza A and B (Figure S4 in Multimedia Appendix 1).

### Establishment of the Final AI-Lab Model

The final model was created using an extended cross-validation approach, specifically 5-fold cross-validation combined with a random grid search for hyperparameter tuning. Its performance was evaluated on both internal testing and external validation cohorts, measuring the AUC and corresponding 95% CIs based on 2000 nonparametric bootstrap samples with replacement. Shapley additive explanations (SHAP) were used to illustrate the contributions of individual variables to each category, using the SHAP Python package. The model’s effectiveness was assessed in both the internal and external validation cohorts. The resulting tool, AI-Lab, was developed as an open-source Python package featuring a user-friendly graphical interface. This web application is freely available, allowing global access without restrictions.

### Ethical Considerations

This study was conducted in accordance with the Declaration of Helsinki and approved by the Medical Ethics Committee of the Affiliated Dazu’s Hospital of Chongqing Medical University approved the study (approval number DZ2024-04-039). It was classified as minimal-risk research involving retrospective analysis of fully deidentified clinical data, and the ethics committee granted a waiver of additional ethical review. Informed consent was waived because the study involved secondary analysis of preexisting, fully anonymized medical records with no possibility of reidentifying individual participants, posed no foreseeable risks to participants’ rights, welfare, or privacy, and the original data collection process obtained appropriate informed consent, including permission for future secondary analyses. All data were fully deidentified prior to analysis, with the removal of personally identifiable information such as names, contact details, and medical identification numbers, and stored on a password-protected, encrypted server accessible only to authorized researchers. Results are reported exclusively in aggregate form to prevent any potential re-identification of individuals. No compensation was provided to participants, as the study involved a retrospective analysis of existing data and did not require direct participant involvement. This study did not include any images or supplementary materials that could potentially identify individual participants; if such materials are included in future studies, explicit written consent will be obtained from identifiable individuals, and consent forms will be submitted as Multimedia Appendices.

## Results

### Patient Characteristics

A total of 2951 patients diagnosed with influenza A virus infection (A+ group), influenza B virus infection (B+ group), or those presenting with influenza-like symptoms but testing negative for both viruses (A–/B– group) between January 1, 2023, and May 31, 2024, were included in the internal testing cohort (Table S2 in Multimedia Appendix 1). This cohort comprised patients from the Affiliated Dazu’s Hospital of Chongqing Medical University and the Affiliated Bishan’s Hospital of Chongqing Medical University. Due to varying incidence rates between influenza A and B, the retrospective cohort included 807 patients in the A+ group, 396 patients in the B+ group, and 1748 patients in the A–/B– group. For the external validation cohort, 3677 patients were sourced from the First Affiliated Hospital of Chongqing Medical University. This cohort included 436 patients in the A+ group, 316 patients in the B+ group, and 2925 patients in the A–/B– group.

### Evaluation of the Diagnostic Performance of Different Models for Influenza A and B Infection in the Internal Testing Cohort

**Figure 1:**
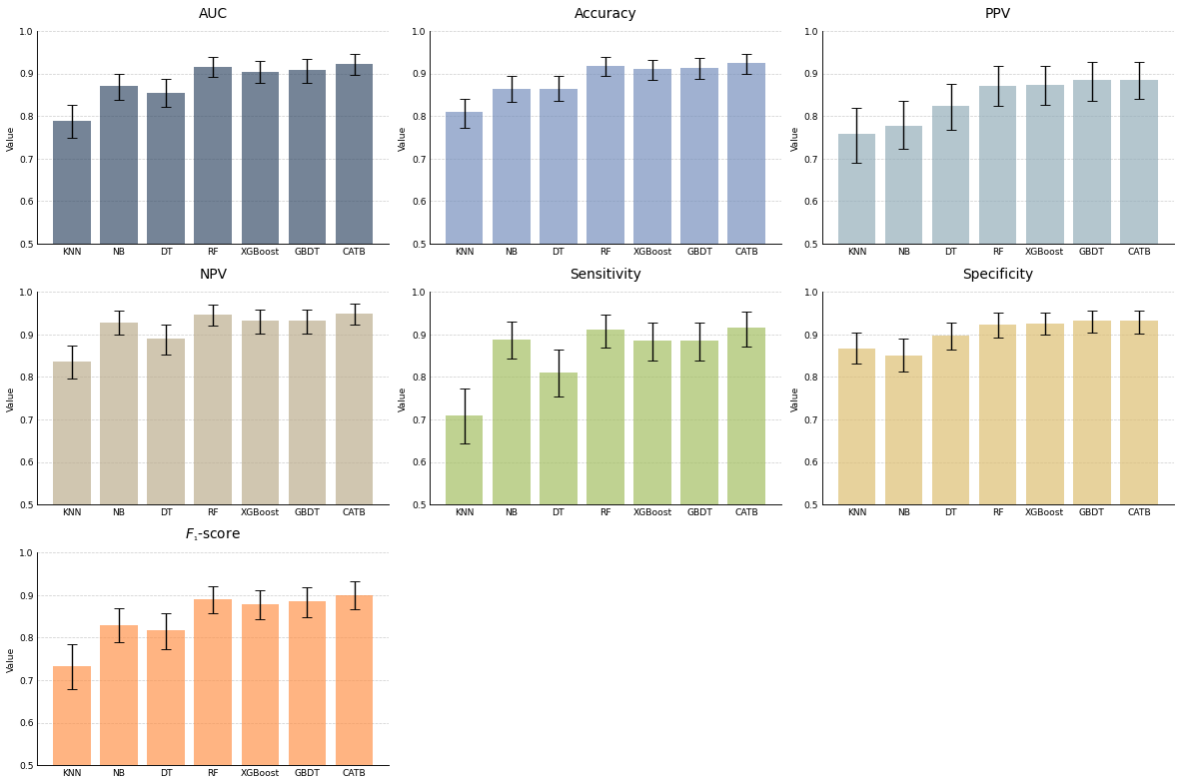
Diagnostic performance of models in A+ and A–/B– groups in the internal testing cohort. AUC: area under the curve; CATB: CatBoost; DT: decision tree; GBDT: gradient-boosting decision tree; KNN: K-nearest neighbors; NB: naïve Bayes; NPV: negative predictive value; PPV: positive predictive value; RF: random forest; XGBoost: extreme gradient boosting.

**Figure 2:**
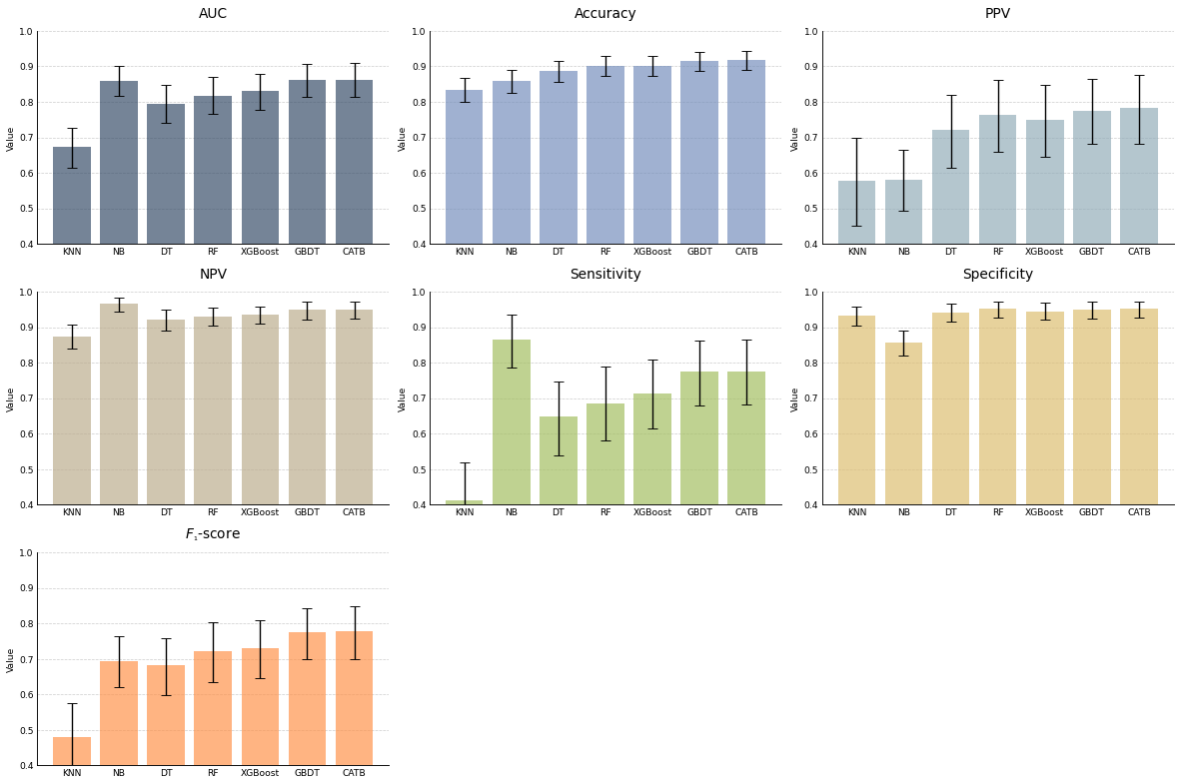
Diagnostic performance of models in B+ and A–/B– groups in the internal testing cohort. AUC: area under the curve; CATB: CatBoost; DT: decision tree; GBDT: gradient-boosting decision tree; KNN: K-nearest neighbors; NB: naïve Bayes; NPV: negative predictive value; PPV: positive predictive value; RF: random forest; XGBoost: extreme gradient boosting.

This study used 7 commonly used medical diagnostic metrics to evaluate the classification performance of different machine learning models optimized through hyperparameter tuning: AUC, accuracy, specificity, sensitivity, positive predictive value (PPV), negative predictive value (NPV), and *F*_1_-score. The diagnostic performance metrics are presented in Figures 1 and 2. In the internal test set, we evaluated 7 machine learning models: KNN, NB, DT, RF, XGBoost, GBDT, and CATB.

For diagnosing influenza A infection, the AUC values for these models were as follows: 0.788 (95% CI 0.749-0.826) for KNN, 0.870 (95% CI 0.839-0.899) for NB, 0.854 (95% CI 0.821-0.887) for DT, 0.916 (95% CI 0.891-0.940) for RF, 0.904 (95% CI 0.877-0.929) for XGBoost, 0.908 (95% CI 0.879-0.934) for GBDT, and 0.923 (95% CI 0.897-0.947) for CATB. For diagnosing influenza B infection, the AUC values were as follows: 0.672 (95% CI 0.614-0.727) for KNN, 0.860 (95% CI 0.817-0.900) for NB, 0.795 (95% CI 0.741-0.848) for DT, 0.818 (95% CI 0.765-0.871) for RF, 0.830 (95% CI 0.778-0.880) for XGBoost, 0.862 (95% CI 0.813-0.907) for GBDT, and 0.863 (95% CI 0.814-0.911) for CATB.

By comparing the performance metrics, the ensemble models, which are RF, XGBoost, GBDT, and CATB, showed superior performance, while KNN and NB exhibited unstable results. Ultimately, CATB achieved the highest AUC of 0.923 (95% CI 0.897-0.947) for distinguishing the A+ group from the A–/B– group and the highest AUC of 0.863 (95% CI 0.814-0.911) for distinguishing the B+ group from the A–/B– group.

In Figure 1, bar graphs are presented, comparing the performance metrics of various classification models: KNN, NB, DT, RF, XGBoost, GBDT, and CATB. The metrics include AUC, accuracy, PPV, NPV, sensitivity, and specificity. The error bars in the chart represent the 95% CI, which was obtained through 2000 nonparametric bootstraps.

In Figure 2, bar graphs are presented, comparing the performance metrics of various classification models: KNN, NB, DT, RF, XGBoost, GBDT, and CATB. The metrics include AUC, accuracy, PPV, NPV, sensitivity, and specificity. The error bars in the chart represent the 95% CI, which was obtained through 2000 nonparametric bootstraps.

### Evaluation of the Diagnostic Performance of Different AI-Lab Models for Influenza A and B Infection in the External Validation Cohort

**Figure 3:**
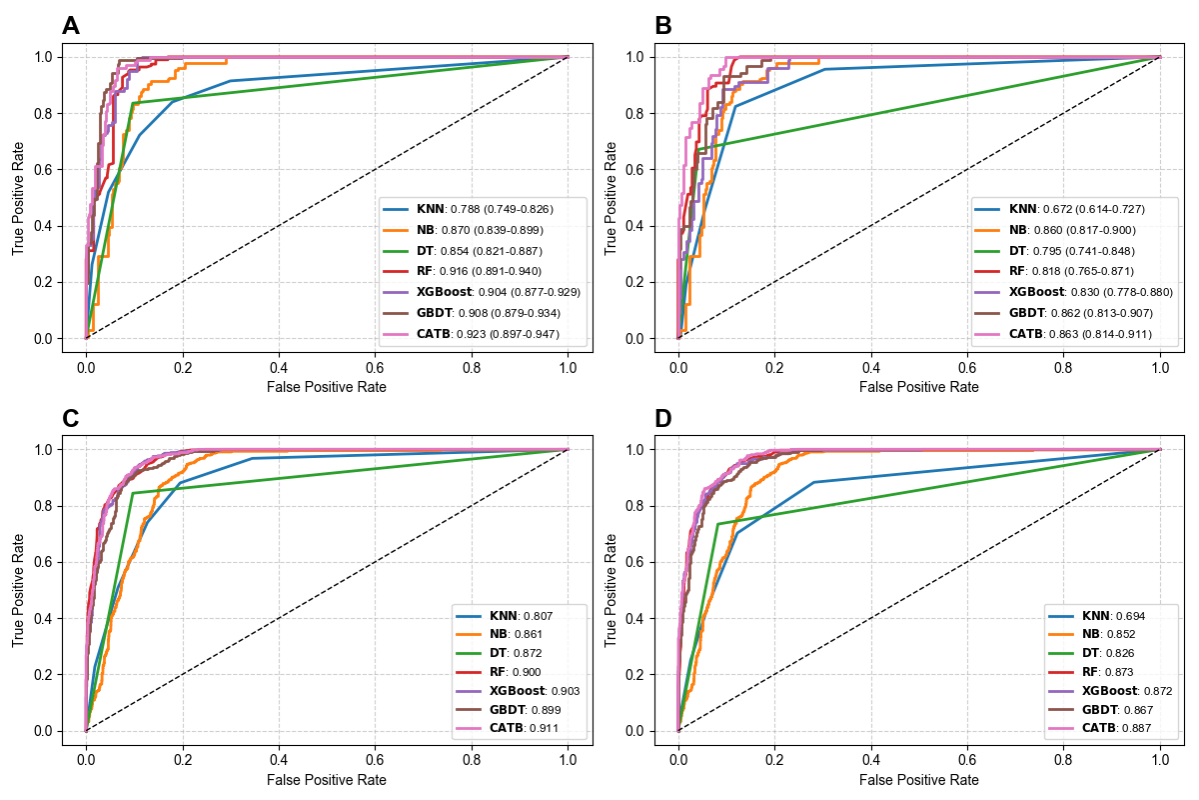
The average receiver operating characteristic (ROC) curves for the 7 models. (A) The average ROC curves for diagnosing influenza A infection in the internal testing cohort. (B) The average ROC curves for diagnosing influenza B infection in the internal testing cohort. (C) The average ROC curves for diagnosing influenza A infection in external validation. (D) The average ROC curves for diagnosing influenza B infection in external validation. CATB: CatBoost; DT: decision tree; GBDT: gradient-boosting decision tree; KNN: K-nearest neighbors; NB: naïve Bayes; RF: random forest; XGBoost: extreme gradient boosting.

To further evaluate the diagnostic performance of different AI-Lab models for influenza A and B infection, we simulated its application in real clinical environments and conducted additional validation using a multicenter external validation cohort. To comprehensively assess the performance of the model across different clinical scenarios, we tested various classification algorithms. In the external validation cohort, the AUC values for diagnosing influenza A infection using KNN, NB, DT, RF, XGBoost, GBDT, and CATB were 0.807, 0.861, 0.872, 0.900, 0.903, 0.899, and 0.911, respectively. For diagnosing influenza B infection, the AUCs were 0.694, 0.852, 0.826, 0.873, 0.872, 0.867, and 0.887. By analyzing these results, we observed that ensemble learning methods (RF, XGBoost, GBDT, and CATB) demonstrated relatively ideal performance, with stable AUC values across different validation sets. In contrast, single models such as KNN, NB, and DT performed noticeably worse, showing significant differences compared to the internal validation results. In the external validation cohort, the CATB-based AI-Lab model achieved the highest AUC values and accuracy: an AUC of 0.911 and an accuracy of 91.3% for differentiating the A+ group from the A–/B– group, and an AUC of 0.887 and an accuracy of 93.9% for distinguishing the B+ group from the A–/B– group (Tables 1 and 2 and Figure 3). Overall, the CATB-based AI-Lab model demonstrated superior performance, particularly in its generalization capability, and was ultimately identified as the optimal model.

**Table 1:** Diagnostic performance metrics of different models for the A+ group and A—/B— group in the external validation cohort.

Classifiers	AUC^a^	Accuracy	PPV^b^	NPV^c^	Sensitivity	Specificity	*F*_1_-score
KNN^d^	0.807	0.855	0.464	0.958	0.741	0.872	0.571
NB^e^	0.861	0.832	0.429	0.982	0.901	0.821	0.581
DT^f^	0.872	0.895	0.565	0.975	0.842	0.903	0.676
RF^g^	0.900	0.904	0.584	0.983	0.894	0.905	0.707
XGBoost^h^	0.903	0.910	0.603	0.983	0.894	0.912	0.720
GBDT^i^	0.899	0.913	0.615	0.981	0.881	0.918	0.725
CATB^j^	0.911	0.913	0.609	0.985	0.908	0.913	0.729

^a^AUC: area under the curve.

^b^PPV: positive predictive value.

^c^NPV: negative predictive value.

^d^KNN: K-nearest neighbors.

^e^NB: naïve Bayes.

^f^DT: decision tree.

^g^RF: random forest.

^h^XGBoost: extreme gradient boosting.

^i^GBDT: gradient-boosting decision tree.

^j^CATB: CatBoost.

**Table 2:** Diagnostic performance metrics of different models for the B+ group and A–/B– group in the external validation cohort.

Classifiers	AUC^a^	Accuracy	PPV^b^	NPV^c^	Sensitivity	Specificity	*F*_1_-score
KNN^d^	0.694	0.889	0.435	0.941	0.453	0.936	0.443
NB^e^	0.852	0.830	0.352	0.984	0.880	0.825	0.503
DT^f^	0.826	0.899	0.489	0.970	0.734	0.917	0.587
RF^g^	0.873	0.937	0.642	0.977	0.794	0.952	0.710
XGBoost^h^	0.872	0.935	0.634	0.977	0.794	0.950	0.705
GBDT^i^	0.867	0.932	0.616	0.976	0.788	0.947	0.692
CATB^j^	0.887	0.939	0.647	0.980	0.823	0.951	0.724

^a^AUC: area under the curve.

^b^PPV: positive predictive value.

^c^NPV: negative predictive value.

^d^KNN: K-nearest neighbors.

^e^NB: naïve Bayes.

^f^DT: decision tree.

^g^RF: random forest.

^h^XGBoost: extreme gradient boosting.

^i^GBDT: gradient-boosting decision tree.

^j^CATB: CatBoost.

### Analysis of Model Interpretability

**Figure 4:**
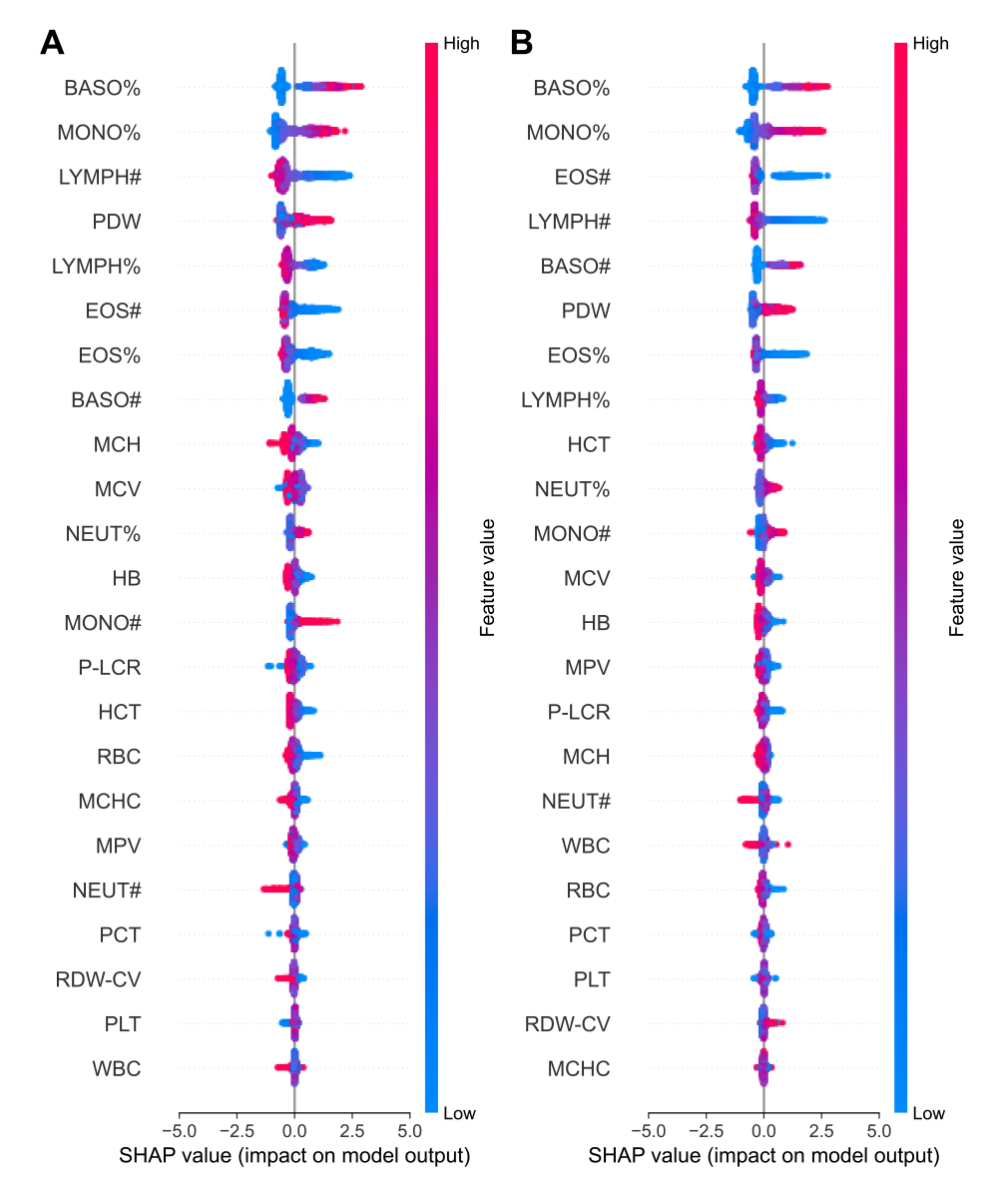
Visualization of Shapley additive explanations (SHAP) values plot for CatBoost, the top-performing machine-learning model. (A) The SHAP values of different blood test metrics in the diagnosis of the A+ group and the A–/B– group reflect their importance for model prediction. (B) The SHAP values of different blood test metrics in the diagnosis of the B+ group and A–/B– group. BASO#: basophil absolute count; BASO%: basophil ratio; EOS#: eosinophil absolute count; EOS%: eosinophil ratio; HB: hemoglobin; HCT: hematocrit; LYMPH#: lymphocyte absolute count; LYMPH%: lymphocyte ratio; MCH: mean corpuscular hemoglobin; MCHC: mean corpuscular hemoglobin concentration; MCV: mean corpuscular volume; MONO#: monocyte absolute count; MONO%: monocyte ratio; MPV: mean platelet volume; NEUT#: neutrophil absolute count; NEUT%: neutrophil ratio; PCT: plateletcrit; PDW: platelet distribution width; P-LCR: large platelet ratio; PLT: platelet count; RBC: red blood cell; RDW-CV: red cell distribution width; WBC: white blood cell count.

SHAP values are an interpretability method in machine learning used to assess the marginal contribution of features to model outputs, explaining “black-box models” from both global and local perspectives [17]. We used the CATB-based AI-Lab model, polynomial classification methods, and improved hyperparameter tuning (optimized through random grid search and 5-fold cross-validation) to construct and evaluate the optimal model, assessing it on both internal test sets and external validation sets. Through SHAP value analysis, we explored the relationships between different features and their impact on the final decision, aiming to understand the model’s decision-making process and investigate the role of laboratory test results in the final diagnosis. When calculating the SHAP value, the key features common to influenza A and influenza B include BASO%, MONO%, LYMPH#, platelet distribution width, LYMPH%, EOS#, and EOS%. In the final 2 CATB models, BASO% and MONO% have the highest discrimination. The relevant results are shown in Figure 4.

## Discussion

### Principal Findings

This study developed and validated a machine learning model for the early identification of adult influenza A and B infections using routine blood parameters. The CATB-based AI-Lab model demonstrated superior performance, achieving AUC values of 0.923 for influenza A and 0.863 for influenza B in the internal test cohort. These results were consistently validated in an external cohort, with AUC values of 0.911 for influenza A and 0.887 for influenza B. The model’s high accuracy and generalization capability highlight its potential as a cost-effective and efficient diagnostic tool for clinical use.

### Comparison to Prior Work

Influenza viruses are classified into 4 major types (A, B, C, and D) based on their nucleoprotein and matrix protein antigens, with types A and B being the most clinically significant [18-22]. Influenza A, known for its high antigenic variability, is a major cause of regional outbreaks and global pandemics [23,24]. In contrast, influenza B exhibits lower antigenic variability and typically causes milder epidemics [25-27]. The symptoms of influenza in adults often differ from those in children, usually presenting as high fever, chills, muscle pain, sore throat, and cough [28-31]. Adults infected with influenza A may experience more severe symptoms, such as difficulty breathing and multiorgan dysfunction, especially in immunocompromised individuals [32]. While influenza B symptoms in adults are generally milder and most patients recover quickly, severe cases can rapidly develop into life-threatening acute respiratory distress syndrome and septic shock [33].

Currently, influenza diagnosis mainly depends on detecting viral nucleic acids and antibodies [34,35]. Although viral isolation and culture have traditionally been regarded as the “gold standard,” these techniques are time-consuming, costly, and technically complex, thus limiting their routine diagnostic application. RT-PCR has emerged as an effective method for nucleic acid detection, offering a sensitivity of 98.5% and a specificity of 100% [36]. However, due to its high cost and time requirements, the application of RT-PCR in routine influenza screening remains limited.

This study emphasizes the development of an efficient and cost-effective diagnostic tool for the early identification of adult influenza A and B infections by combining routine blood parameters with machine learning algorithms. The results indicate that ensemble learning methods, particularly the CATB-based AI-Lab model, outperform other machine learning techniques, demonstrating high accuracy and strong generalization capabilities across different clinical settings. In the internal test cohort, the CATB-based AI-Lab model achieved AUC values of 0.923 for influenza A and 0.863 for influenza B, with consistent performance in the external validation cohort (AUC 0.911 for influenza A and 0.887 for influenza B). These findings highlight the ability of ensemble techniques to capture complex patterns in the data, enhancing predictive accuracy and robustness [37,38].

SHAP analysis further revealed the importance of specific hematological markers, such as BASO%, MONO%, LYMPH#, platelet distribution width, LYMPH%, EOS#, and EOS%, in predicting influenza A and B infections. Notably, these markers differ from those identified in pediatric studies, underscoring the need for age-specific diagnostic models. The integration of the machine learning model into clinical practice through the AI-Lab tool, a user-friendly platform supporting automatic, manual, and batch data input, provides a practical solution for rapid and accurate influenza screening. This tool has the potential to support timely clinical decision-making, ultimately improving patient outcomes.

### Limitations

Despite its promising results, this study has several limitations. First, the reliance on routine blood parameters may limit the model’s applicability across diverse populations and health care settings, as hematological profiles can vary significantly. Second, while the CATB-based AI-Lab model demonstrated excellent performance, further validation using larger and more diverse datasets is necessary to confirm its generalizability [39]. Third, the study focused exclusively on influenza A and B, excluding other respiratory pathogens that may present with similar clinical features. Future research should explore the integration of additional clinical variables (eg, imaging data and comorbidities) and extend the application of these models to other respiratory infectious diseases, such as RSV or SARS-CoV-2, to enhance their diagnostic utility.

### Conclusions

This study demonstrates that machine learning models, particularly the CATB-based AI-Lab model, can effectively predict adult influenza A and B infections using routine blood parameters. The model achieved high accuracy in both internal testing and external validation cohorts, with AUC values of 0.923 and 0.863 for influenza A and B in the internal cohort and 0.911 and 0.887 in the external cohort, respectively. These results highlight the potential of the model as a cost-effective and efficient diagnostic tool for clinical use. However, the findings are limited to influenza A and B, and further validation in diverse populations and health care settings is necessary to confirm generalizability.

## Appendix

Multimedia Appendix 1 Clinical characterization and machine learning workflows for influenza A/B virus infections.

## Abbreviations

AI: artificial intelligence
AI-Lab: Artificial Intelligence Prediction of Influenza A and B
AUC: area under the curve
BASO%: basophils ratio
CATB: CatBoost
DT: decision tree
EOS#: eosinophil absolute count
EOS%: eosinophil ratio
GBDT: gradient-boosting decision tree
KNN: K-nearest neighbors
LYMPH#: lymphocyte absolute count
LYMPH%: lymphocyte ratio
MONO%: monocyte ratio
NB: naïve Bayes
NPV: negative predictive value
PPV: positive predictive value
RF: random forest
RT-PCR: reverse transcriptase–polymerase chain reaction
SHAP: Shapley additive explanations
XGBoost: extreme gradient boosting

## Data Availability

The datasets generated or analyzed during this study are not publicly available due to the data privacy protection policies but are available from the corresponding author on reasonable request.
