# Supplementary material for: Evaluation of a Machine Learning Model Based on Laboratory Parameters for the Prediction of Influenza A and B in Chongqing, China: Multicenter Model Development and Validation Study
Source: J Med Internet Res. 2025 May 15;27:e67847. doi: 10.2196/67847 (PMC12123241; doi:10.2196/67847)
Supplement: Multimedia Appendix 1 [file jmir_v27i1e67847_app1.docx]

**
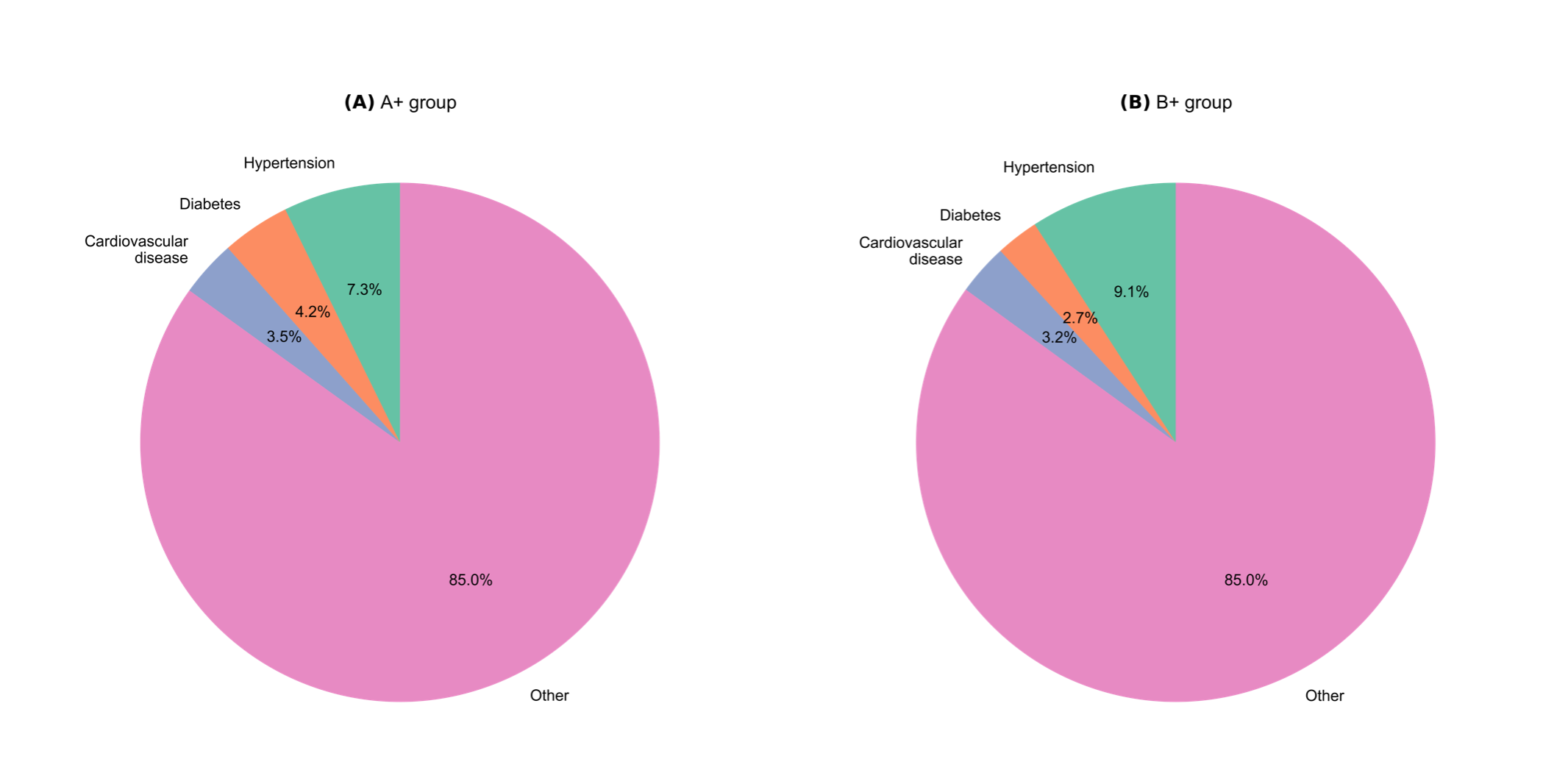
**

**Figure S1. The patient diagnosed with influenza A virus infection (A+ group) and influenza B virus infection (B+ group) demographics and pre-existing health conditions.** The proportions of patients with hypertension, diabetes, cardiovascular diseases, and those without health conditions in the A+ group and B+ group.


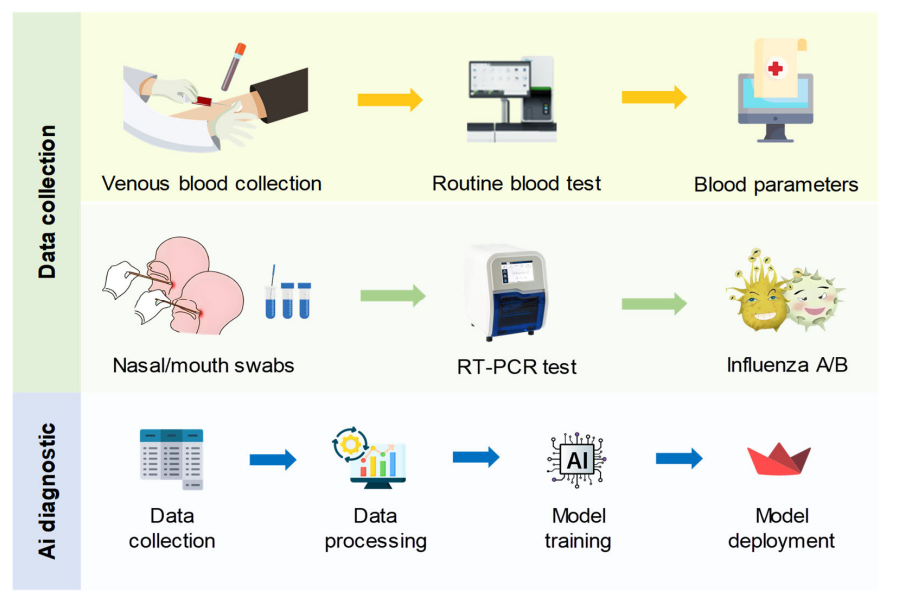


**Figure S2. Flowchart of the study.** The rose column and the green column show the collection of the patient’s blood cell parameters and the process of RT-PCR testing for influenza A/B. The overall flow chart of the predictive model Workflow diagram of our Ai diagnosis framework. The diagram illustrates the process of dataset construction through the collection of routine blood parameters and the use of RT-PCR for influenza virus detection. In the data preprocessing stage, the collected data undergoes preprocessing to prepare it for model training. Subsequently, multiple machine learning methods are utilized to construct the model, and the model with the best performance is selected.

**
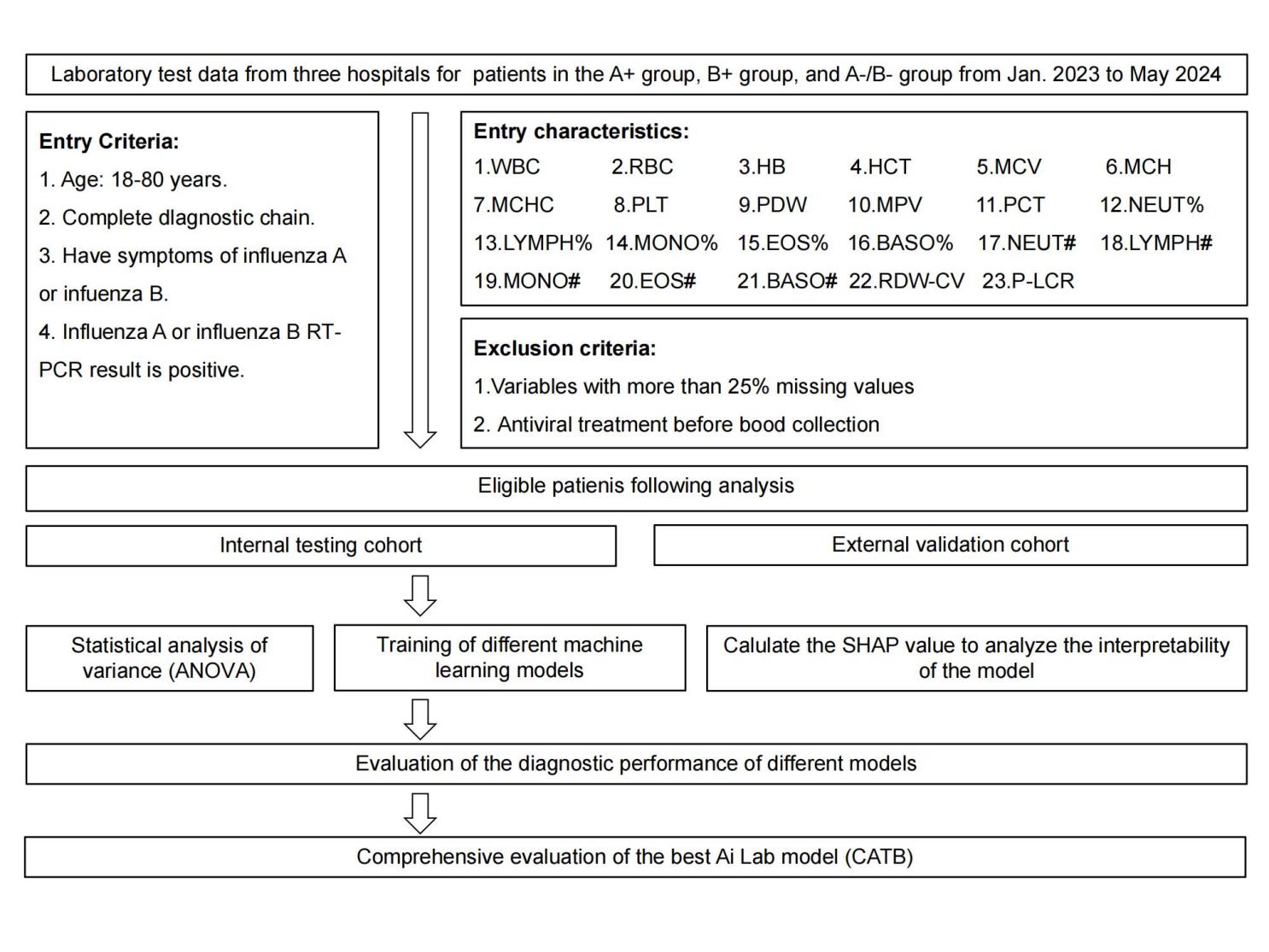
**

**Figure S3. Flowchart of machine learning to explore data.** The process of the model is divided into five stages: analysis of eligible patients, training of different machine learning models, calculation of SHAP values to analyze the interpretability of the model, evaluation of the diagnostic performance of different models, and comprehensive evaluation of the best Ai Lab model (CATB).

**
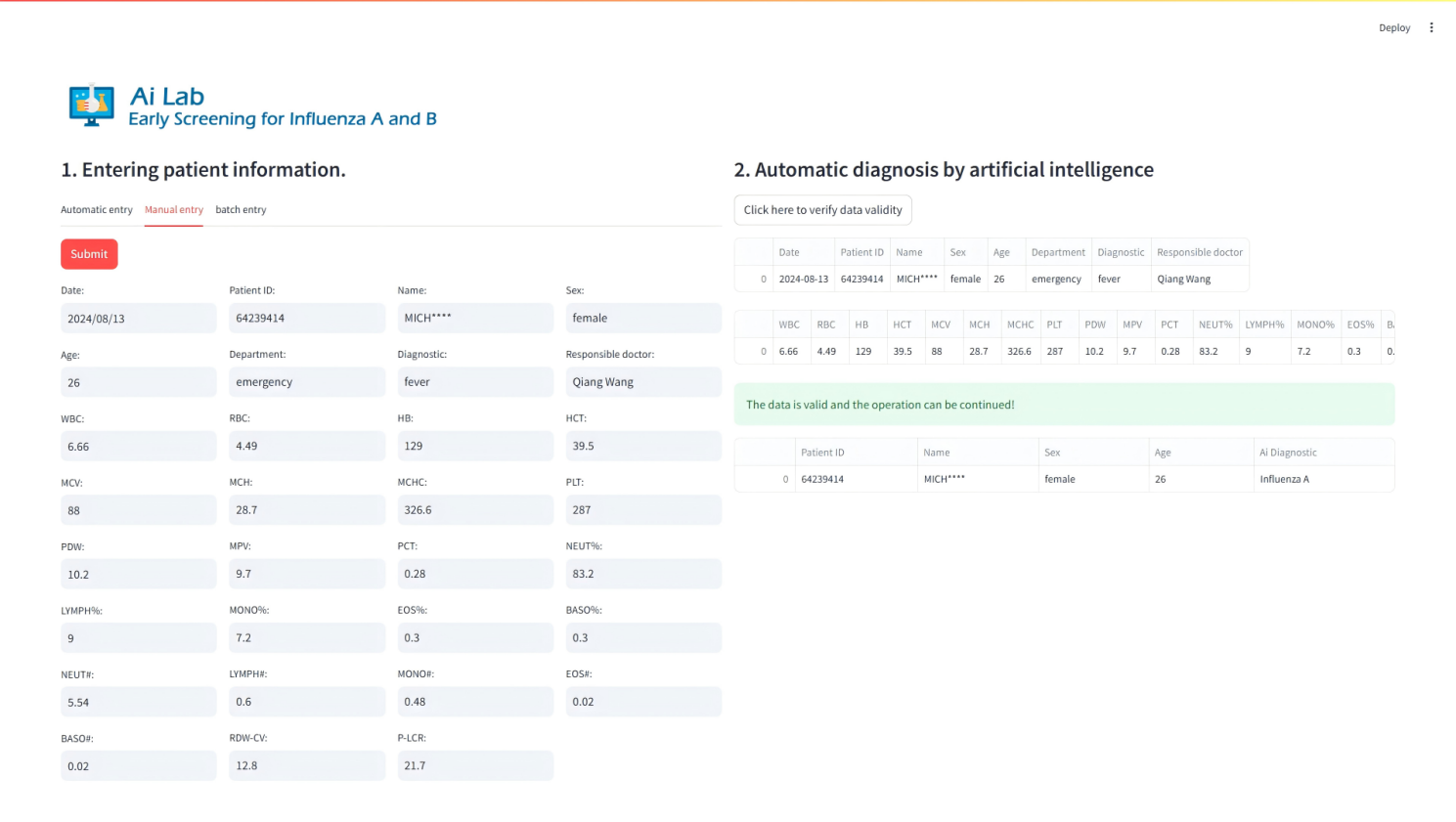
**

**Figure S4. Demonstration of the system interface operation.** It consists of two parts: entering patient information and automatic diagnosis. After the system performs the automatic diagnosis, a specific diagnostic report is generated, including the patient’s personal details and all the parameter values used for the diagnosis, aiding in documenting the disease progression.

**Table S1.** Setting of the optimal hyperparameter search range for randomized grid search.

| **Classifier** | **Model** | **Parameters** |
| --- | --- | --- |
| **KNN** | KNeighborsClassifier() | "n_neighbors": range(1, 31),  "weights": ["uniform", "distance"],  "metric": ["euclidean", "manhattan"], |
| **NB** | GaussianNB() | - |
| **DT** | DecisionTreeClassifier() | "max_depth": [None, 10, 20, 30, 40, 50],  "min_samples_split": [2, 5, 10],  "criterion": ["gini", "entropy"], |
| **RF** | RandomForestClassifier() | "n_estimators": [100, 200, 300],  "max_depth": [10, 20, 30],  "min_samples_split": [2, 5], |
| **XGB** | XGBClassifier() | "max_depth": [3, 5, 7],  "learning_rate": [0.01, 0.1, 0.2],  "n_estimators": [100, 200],  "subsample": [0.8, 0.9], |
| **GBDT** | GradientBoostingClassifier() | "n_estimators": [100, 200],  "learning_rate": [0.01, 0.1], |
| **CATB** | CatBoostClassifier() | "iterations": [500, 1000, 2000],  "learning_rate": [0.001, 0.01, 0.1], |

**Table S2** Baseline characteristics and variables are shown for the four different diseases categories (influenza A+, influenza B+, influenza A+/B+ and influenza A-/B-) in the internal testing cohort.

| **Variables** | **Type** | **A+ group (n = 807)** | **B+ group (n = 396)** | **A+/B+ group (n = 1203)** | **A-/B- group (n = 1748)** | ***p*-value** |
| --- | --- | --- | --- | --- | --- | --- |
| Gender | Male | 385 | 213 | 598 | 1032 | - |
|  | Feamle | 422 | 183 | 605 | 716 | - |
| Age | Mean (std) | 36.050 (18.169) | 36.620 (14.848) | 41.567 (15.659) | 44.798 (13.845) | <0.001* |
|  | Median [IQR] | 28.000 [24.000-38.000] | 31.000 [26.000-45.000] | 38.000 [28.000-53.000] | 45.000 [34.000-55.000] |  |
| WBC | Mean (std) | 6.375 (2.148) | 6.289 (2.568) | 6.307 (1.963) | 6.286 (1.708) | 0.815 |
|  | Median [IQR] | 6.040 [4.875-7.615] | 5.910 [4.515-7.380] | 6.035 [5.000-7.340] | 6.030 [5.105-7.210] |  |
| RBC | Mean (std) | 4.458 (0.562) | 4.508 (0.705) | 4.724 (0.588) | 4.875 (0.510) | <0.001* |
|  | Median [IQR] | 4.440 [4.160-4.810] | 4.560 [4.150-4.955] | 4.700 [4.380-5.110] | 4.825 [4.500-5.210] |  |
| HB | Mean (std) | 131.517 (15.485) | 133.160 (20.420) | 141.761 (18.100) | 147.621 (15.760) | <0.001* |
|  | Median [IQR] | 131.000 [122.000-143.500] | 133.000 [122.000-145.000] | 142.000 [130.000-155.000] | 147.000 [137.000-159.000] |  |
| HCT | Mean (std) | 39.807 (4.307) | 40.222 (5.869) | 42.796 (4.927) | 44.521 (4.017) | <0.001* |
|  | Median [IQR] | 39.800 [36.950-42.700] | 40.300 [37.050-43.800] | 43.000 [39.800-46.400] | 44.650 [41.800-47.600] |  |
| MCV | Mean (std) | 89.690 (6.032) | 89.607 (5.176) | 90.873 (5.722) | 91.614 (5.597) | 0.007 |
|  | Median [IQR] | 89.800 [87.100-92.300] | 89.800 [87.200-92.000] | 91.350 [88.200-93.800] | 92.100 [89.100-94.600] |  |
| MCH | Mean (std) | 29.621 (2.248) | 29.643 (1.923) | 30.093 (2.213) | 30.376 (2.216) | 0.008 |
|  | Median [IQR] | 29.800 [28.900-30.700] | 30.000 [29.100-30.600] | 30.200 [29.225-31.200] | 30.500 [29.500-31.500] |  |
| MCHC | Mean (std) | 330.139 (9.852) | 330.755 (8.860) | 330.859 (9.985) | 331.153 (10.266) | 0.547 |
|  | Median [IQR] | 330.300 [325.700-335.650] | 331.200 [326.450-336.500] | 331.000 [325.000-337.000] | 331.000 [324.750-337.000] |  |
| PLT | Mean (std) | 206.046 (58.720) | 203.025 (68.844) | 223.817 (63.547) | 235.366 (61.161) | <0.001* |
|  | Median [IQR] | 200.000 [166.500-239.000] | 195.000 [160.000-243.000] | 219.000 [180.000-259.750] | 231.000 [195.000-267.250] |  |
| PDW | Mean (std) | 12.954 (2.755) | 13.610 (3.144) | 12.907 (2.572) | 12.724 (2.308) | 0.127 |
|  | Median [IQR] | 12.500 [10.650-15.900] | 13.300 [10.850-16.300] | 12.200 [11.000-15.000] | 12.100 [11.200-13.700] |  |
| MPV | Mean (std) | 10.260 (1.147) | 10.463 (1.290) | 10.484 (1.080) | 10.572 (0.984) | 0.080 |
|  | Median [IQR] | 10.000 [9.500-11.000] | 10.200 [9.650-11.200] | 10.300 [9.800-11.100] | 10.400 [9.900-11.100] |  |
| PCT | Mean (std) | 0.208 (0.053) | 0.208 (0.066) | 0.232 (0.059) | 0.246 (0.055) | <0.001* |
|  | Median [IQR] | 0.200 [0.170-0.240] | 0.200 [0.170-0.250] | 0.230 [0.190-0.270] | 0.240 [0.210-0.280] |  |
| NEUT% | Mean (std) | 72.011 (10.504) | 69.639 (11.356) | 62.637 (11.643) | 57.480 (8.691) | <0.001* |
|  | Median [IQR] | 74.000 [65.750-79.900] | 70.300 [62.050-78.500] | 61.220 [54.410-71.175] | 57.140 [52.000-63.155] |  |
| LYMPH% | Mean (std) | 16.708 (9.212) | 18.783 (9.349) | 27.553 (11.604) | 33.678 (8.047) | <0.001* |
|  | Median [IQR] | 14.300 [9.700-21.600] | 17.300 [11.250-25.850] | 29.300 [18.300-36.100] | 33.600 [28.575-38.255] |  |
| MONO% | Mean (std) | 10.168 (3.351) | 10.183 (3.372) | 7.830 (3.166) | 6.400 (1.964) | <0.001* |
|  | Median [IQR] | 10.000 [7.700-12.150] | 9.900 [7.800-12.500] | 7.000 [5.600-9.500] | 6.200 [5.100-7.400] |  |
| EOS% | Mean (std) | 0.820 (1.119) | 0.882 (1.341) | 1.852 (1.850) | 2.466 (1.902) | <0.001* |
|  | Median [IQR] | 0.400 [0.100-1.200] | 0.300 [0.100-1.300] | 1.400 [0.600-2.500] | 1.900 [1.200-3.110] |  |
| BASO% | Mean (std) | 0.293 (0.178) | 0.304 (0.237) | 0.135 (0.199) | 0.035 (0.113) | <0.001* |
|  | Median [IQR] | 0.300 [0.200-0.400] | 0.200 [0.100-0.400] | 0.040 [0.000-0.200] | 0.000 [0.000-0.040] |  |
| NEUT# | Mean (std) | 4.699 (1.973) | 4.453 (2.175) | 4.029 (1.741) | 3.679 (1.405) | <0.001* |
|  | Median [IQR] | 4.470 [3.400-5.845] | 3.990 [2.900-5.340] | 3.640 [2.772-4.870] | 3.380 [2.690-4.283] |  |
| LYMPH# | Mean (std) | 0.977 (0.493) | 1.111 (0.720) | 1.670 (0.769) | 2.061 (0.576) | <0.001* |
|  | Median [IQR] | 0.850 [0.620-1.240] | 1.000 [0.630-1.390] | 1.690 [1.080-2.170] | 2.005 [1.690-2.392] |  |
| MONO# | Mean (std) | 0.629 (0.249) | 0.623 (0.270) | 0.483 (0.224) | 0.395 (0.143) | <0.001* |
|  | Median [IQR] | 0.590 [0.450-0.770] | 0.610 [0.440-0.765] | 0.440 [0.330-0.600] | 0.370 [0.290-0.480] |  |
| EOS# | Mean (std) | 0.052 (0.081) | 0.052 (0.080) | 0.112 (0.116) | 0.149 (0.119) | <0.001* |
|  | Median [IQR] | 0.020 [0.010-0.065] | 0.020 [0.010-0.070] | 0.080 [0.030-0.150] | 0.120 [0.070-0.190] |  |
| BASO# | Mean (std) | 0.019 (0.014) | 0.018 (0.015) | 0.008 (0.013) | 0.002 (0.007) | <0.001* |
|  | Median [IQR] | 0.020 [0.010-0.020] | 0.010 [0.010-0.025] | 0.000 [0.000-0.010] | 0.000 [0.000-0.000] |  |
| RDW-CV | Mean (std) | 12.802 (1.267) | 13.158 (1.795) | 13.035 (1.218) | 13.094 (1.000) | 0.291 |
|  | Median [IQR] | 12.500 [12.200-13.100] | 12.700 [12.200-13.350] | 12.800 [12.300-13.400] | 12.950 [12.500-13.500] |  |
| P-LCR | Mean (std) | 26.965 (8.629) | 28.485 (9.545) | 28.653 (7.795) | 29.324 (6.852) | 0.063 |
|  | Median [IQR] | 25.100 [20.900-32.900] | 26.100 [22.350-34.150] | 27.700 [23.200-33.575] | 28.450 [24.600-33.600] |  |

*p*-values are adjusted using the FDR method.

^*^shows the signifcant diferences between the A+/B+ group and A-/B- group.

A+ group: patients with influenza A virus infection, B+ group: patients with influenza B virus infection, A+/B+ group: patients with influenza A or B virus infection, A-/B- group: patients with influenza-like symptoms who were negative for both influenza A and B viruses.

Absolute numbers and percentages are used for categorical variables and mean and standard deviation are used for continuous variables.
